# Heterogeneity of Fractional Anisotropy and Mean Diffusivity Measurements by In Vivo Diffusion Tensor Imaging in Normal Human Hearts

**DOI:** 10.1371/journal.pone.0132360

**Published:** 2015-07-15

**Authors:** Laura-Ann McGill, Andrew D. Scott, Pedro F. Ferreira, Sonia Nielles-Vallespin, Tevfik Ismail, Philip J. Kilner, Peter D. Gatehouse, Ranil de Silva, Sanjay K. Prasad, Archontis Giannakidis, David N. Firmin, Dudley J. Pennell

**Affiliations:** 1 NIHR Cardiovascular Biomedical Research Unit, Royal Brompton Hospital, Sydney Street, London, SW3 6NP, United Kingdom; 2 National Heart and Lung Institute, Imperial College, London, United Kingdom; Gent University, BELGIUM

## Abstract

**Background:**

Cardiac diffusion tensor imaging (cDTI) by cardiovascular magnetic resonance has the potential to assess microstructural changes through measures of fractional anisotropy (FA) and mean diffusivity (MD). However, normal variation in regional and transmural FA and MD is not well described.

**Methods:**

Twenty normal subjects were scanned using an optimised cDTI sequence at 3T in systole. FA and MD were quantified in 3 transmural layers and 4 regional myocardial walls.

**Results:**

FA was higher in the mesocardium (0.46 ±0.04) than the endocardium (0.40 ±0.04, p≤0.001) and epicardium (0.39 ±0.04, p≤0.001). On regional analysis, the FA in the septum was greater than the lateral wall (0.44 ±0.03 vs 0.40 ±0.05 p = 0.04). There was a transmural gradient in MD increasing towards the endocardium (epicardium 0.87 ±0.07 vs endocardium 0.91 ±0.08×10^-3 ^mm^2^/s, p = 0.04). With the lateral wall (0.87 ± 0.08×10^-3 ^mm^2^/s) as the reference, the MD was higher in the anterior wall (0.92 ±0.08×10^-3 ^mm^2^/s, p = 0.016) and septum (0.92 ±0.07×10^-3 ^mm^2^/s, p = 0.028). Transmurally the signal to noise ratio (SNR) was greatest in the mesocardium (14.5 ±2.5 vs endocardium 13.1 ±2.2, p<0.001; vs epicardium 12.0 ± 2.4, p<0.001) and regionally in the septum (16.0 ±3.4 vs lateral wall 11.5 ± 1.5, p<0.001). Transmural analysis suggested a relative reduction in the rate of change in helical angle (HA) within the mesocardium.

**Conclusions:**

In vivo FA and MD measurements in normal human heart are heterogeneous, varying significantly transmurally and regionally. Contributors to this heterogeneity are many, complex and interactive, but include SNR, variations in cardiac microstructure, partial volume effects and strain. These data indicate that the potential clinical use of FA and MD would require measurement standardisation by myocardial region and layer, unless pathological changes substantially exceed the normal variation identified.

## Introduction

Cardiac diffusion tensor imaging (cDTI) offers novel characteristation of myocardial microstructures [1–5]. Recent technical advances in magnetic resonance (MR) hardware, combined with sequence development, have enabled reproducible in-vivo cDTI of the human heart [6–8]. The ability to interrogate the microarchitecture non-invasively has the potential to advance our understanding of diseases, such as hypertrophic cardiomyopathy, where the myocardium is reported to show disarray [9–12].

cDTI exploits the tissue specific nature of water diffusion in biological tissues, which occurs preferentially along the length of cellular structures [13,14]. From the diffusion tensor, quantitative parameters such as mean diffusivity (MD), fractional anisotropy (FA) and the helical angle (HA) can be calculated [15]. Collectively these describe the freedom of myocardial water movement, the organisation of myocardial microarchitecture, and the orientation of myocytes. Ex-vivo cDTI studies have demonstrated a close correlation between transmural DTI results and histological appearances [16–19]. Similar work in-vivo has been limited by the inherently poor signal to noise ratio (SNR) of the technique, and the challenge of detecting diffusion on a scale of μm, in the presence of bulk cardiac motion (on a scale of mm). Interpretation of quantitative in-vivo cDTI parameters, derived from a monopolar sequence, is further complicated by the impact of myocardial strain on the diffusion tensor [20]. Comparison with data acquired ex-vivo and using strain insensitive in-vivo acquisitions (monopolar ‘sweet spot’ or bipolar techniques) therefore help to contextualise results [21–22]. Moreover, in-vivo cDTI measurements are thought to include a contribution from microvascular perfusion [23–25], which in diseased myocardium may affect DTI parameters unpredictably. Further research addressing these issues is therefore required before cDTI can be clinically implemented.

Recent work in our department has sought to establish the optimal diffusion weighting for both the diffusion encoded (b_main_) and the reference data (b_ref_), with respect to myocardial characterisation with cDTI [26]. We found that elevating b_main_ from 350s/mm^2^, as adopted by previous studies [6,7], to 750s/mm^2^ provided enhanced transmural image quality. Additionally we have proposed that increasing b_ref_ from ≈0s/mm^2^ to 150s/mm^2^ minimises the contribution from microvascular perfusion [26].

Although some ex vivo cDTI studies have addressed in-homogeneity in anisotropy and diffusivities [27–29], most have assumed that these measures are homogeneous; there also remains a paucity of in-vivo data in the normal heart on which to compare normal with diseased myocardium, to determine whether appreciable abnormalities exist. In this study we describe our observations of the heterogeneity of quantitative transmural and regional cDTI, in a healthy cohort of volunteers with an optimised in-vivo sequence.

## Methods

### In-vivo Imaging Sequence

Twenty healthy volunteers (average age 32 [range 22–57], 15 male) were recruited, including data from 10 volunteers who contributed to our previous study [26]. This study was approved by the NRES Committee South East Coast Surrey (REC reference 10/H0701/112), all subjects gave written consent. Images were acquired using a 3T scanner (Magnetom Skyra, Siemens AG Healthcare Sector, Erlangen, Germany) with an anterior 18 element matrix coil and 8–12 elements of a matrix spine coil. Initial localisation images were acquired to determine the short axis of the left ventricle (LV). A mid ventricular retro-gated cine sequence, with a temporal resolution of 40ms, was then obtained to establish the timing of the subject specific end systolic pause. cDTI was acquired in a single, mid LV short axis slice during the systolic pause. Breath-hold cDTI was performed with a monopolar, diffusion-weighted stimulated echo acquisition mode (DW-STEAM) EPI sequence [6,7]. The EPI echo train was reduced with zonal excitation in the phase encode direction and GRAPPA parallel imaging [30]. The sequence is gated to every other cardiac cycle and makes the assumption that the heart position is identical on consecutive cycles. As stated above, diffusion weighting was set at b_main_ = 750s/mm^2^ and the b_ref_ was 150s/mm^2^ for the separate reference data [2,26]. A minimum of eight averages were acquired at b_main_ and one at b_ref_, with 6 diffusion encoding directions in each case. In each breath hold, 2 sets of calibration data were acquired (GRAPPA reference and EPI phase correction data, 4 cardiac cycles), followed by the standard reference images with minimal diffusion weighting (b = 15s/mm^2^, 2 cardiac cycles, b_main_ only), and finally data in each of the 6 diffusion encoding directions at b_main_ or b_ref_ (12 cardiac cycles). Each breath hold was approximately 18s long and the total scan duration was approximately 20mins. The echo time (TE) was set at 24ms and the remaining sequence parameters were as described previously [6,7], with fat saturation, repetition time (TR) 2 cardiac cycles, field of view 360x135mm^2^, slice thickness 8mm, acquired resolution 2.8x2.8mm^2^, reconstructed resolution 1.4x1.4mm^2^, GRAPPA factor 2, echo train length 24 and echo train duration 13ms.

To examine the impact of resolution on our results, two volunteers were scanned a second time with a higher resolution sequence. The acquired resolution was 1.9 x 1.9 x 6mm with a TE of 37ms, an echo train length of 33 an echo train duration of 24ms, and a field of view of 349x125mm^2^. In order to compensate for the reduced signal to noise ratio at the higher spatial resolution TR was increased to 4 cardiac cycles to allow additional T1 recovery between images and a SENSE reconstruction was used. A minimum of 8 averages were acquired with 6 diffusion encoding directions, the diffusion weighting of b_main_ and b_ref_ were unchanged, but the standard reference images had an increased diffusion weighted of 72s/mm^2^. Breath holds were very long, averaging 40secs, and scan time was 30mins. As only 2 individuals were scanned at this resolution, no quantitative analysis was performed, and parameter maps for HA, MD, FA & SNR results are presented for qualitative assessment.

### Diffusion tensor analysis

All cDTI was post-processed using custom-built software developed in house using MATLAB (Mathworks, MA, USA). All frames were analysed visually and those corrupted by motion were rejected. All images per subject were co-registered with a rapid multi-resolution rigid registration algorithm [31]. A rank 2 diffusion tensor was generated for each voxel and the eigensystem (eigenvalues [e1, e2 and e2] and eigenvectors [$\hat{e_{1}},\hat{e_{2},}\hat{e_{3}}$]) were then calculated for each tensor [32]. The dependence of the b-value on the heart rate was accounted for on a beat-to-beat basis in the tensor calculation. Quantitative maps of FA and MD were calculated from the eigensystem [33]. Mean intravoxel myocyte orientation was assumed to be represented by $\hat{e_{1}}$, from which the myocardial HA was calculated [34]. HA gradient was calculated as described in Lombaert et al. [35], by drawing radial lines from the centre through the myocardium and using a linear regression of HA with transmural depth. The acquisition SNR before averaging was measured using the multiple acquisition method described in Reeder et al. [36], applied to the standard reference images acquired in every b_main_ breath hold (b = 15s/mm^2^). For quantitative analysis, the myocardium was divided into 3 transmural layers (endocardium, mesocardium and epicardium) using regions of interest placed at one-third and two-thirds through the myocardial wall. The myocardium was also analysed in 4 regional full thickness segments (septal, anterior, lateral and inferior). All cDTI analysis was performed by a single observer.

### Statistical analysis

The eigenvalues, MD and FA values were analysed globally, transmurally, and by segment. All values were found to be normally distributed via a visual assessment of the distributions plotted as a histogram, and are therefore shown as mean ±standard deviation (SD). Statistical comparison was first performed using a 1-way repeated measures ANOVA followed by paired t-testing between variables using the Bonferroni correction for multiple tests. The significance level was set at p<0.05. Due to the transmural variation in HA, values of HA gradient were only analysed globally.

## Results

cDTI was successfully performed in all subjects. The average subject RR interval and trigger times were 917 ±187ms and 342 ±91ms respectively.

### Helical Angle

**Fig 1:**
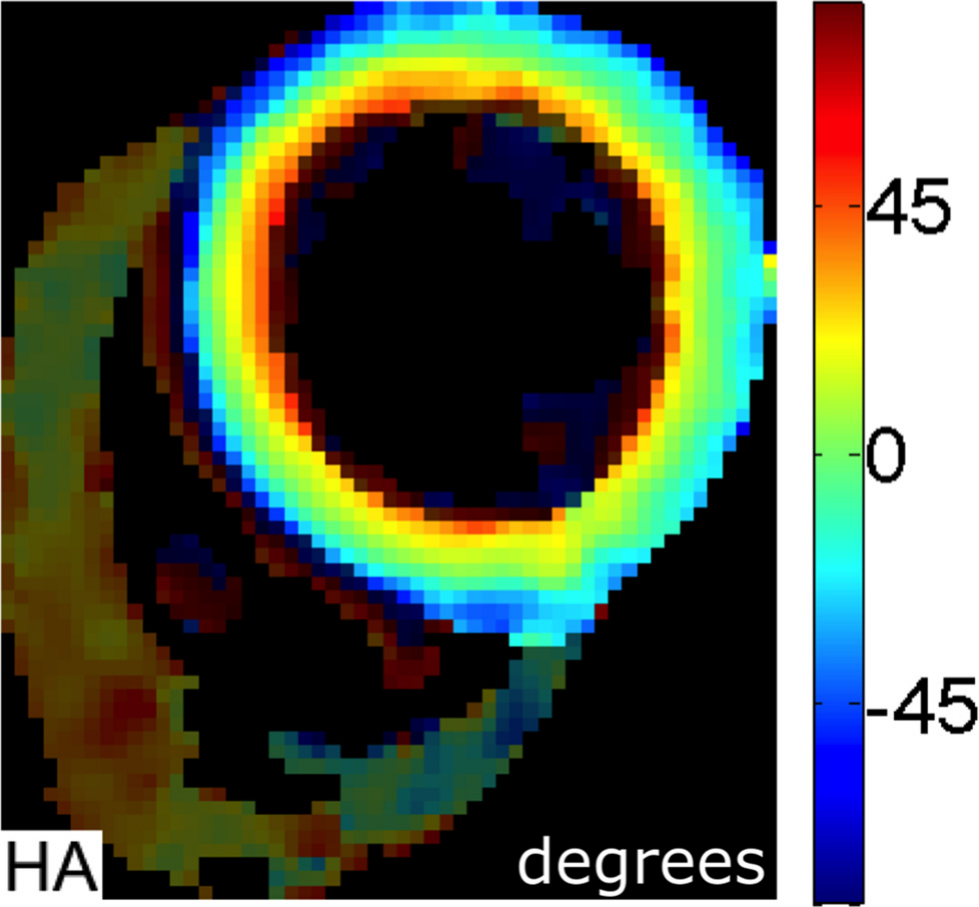
Example of Helical Angle (HA) map. This shows a smooth progression from a left-handed helical pattern in the epicardium (blue) to a circumferential orientation in the mesocardium (green) and right-handed helical pattern in the endocardium (red).

**Fig 2:**
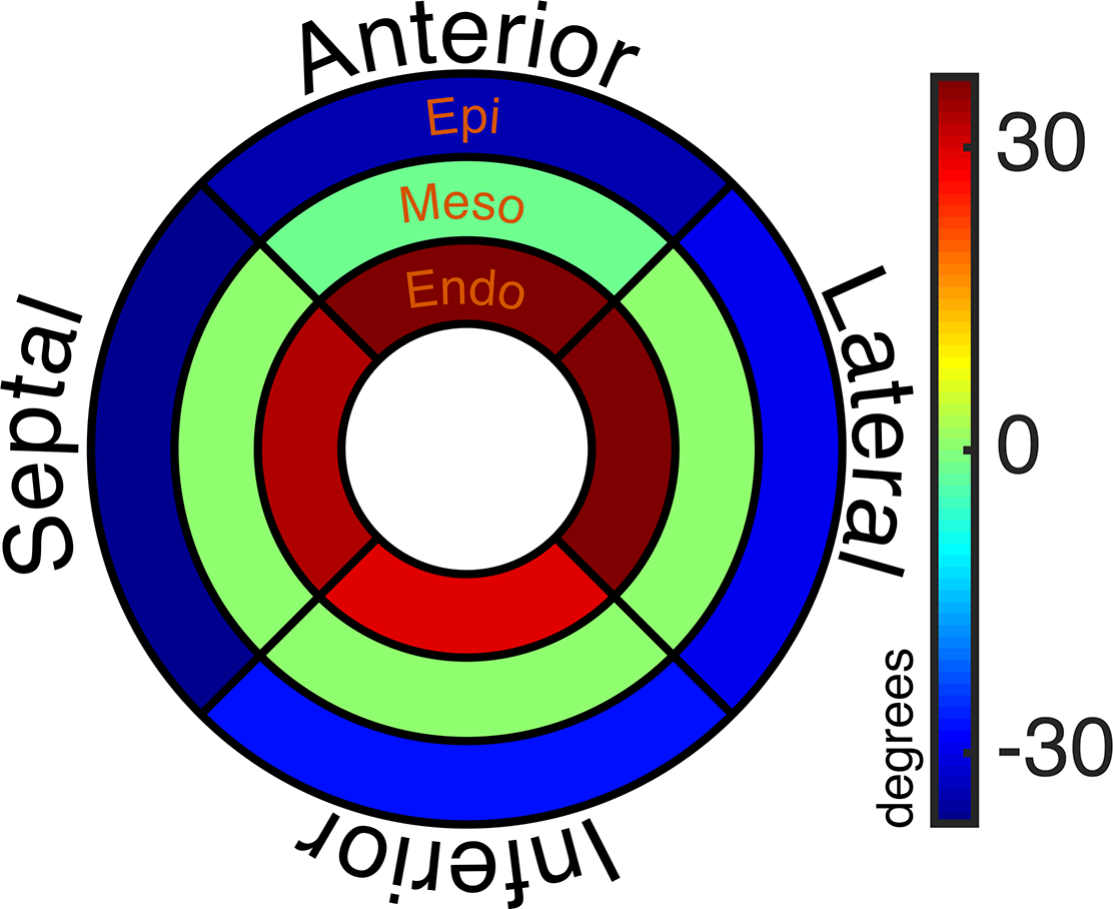
Colour coded Bullseye plot of mean helical angle values per transmural layer and regional wall. The inner, middle and outer rings represent the endo, meso and epicardium respectively. The upper, right, lower and left segments represent the anterior, lateral, inferior and septal walls respectively.

**Fig 3:**
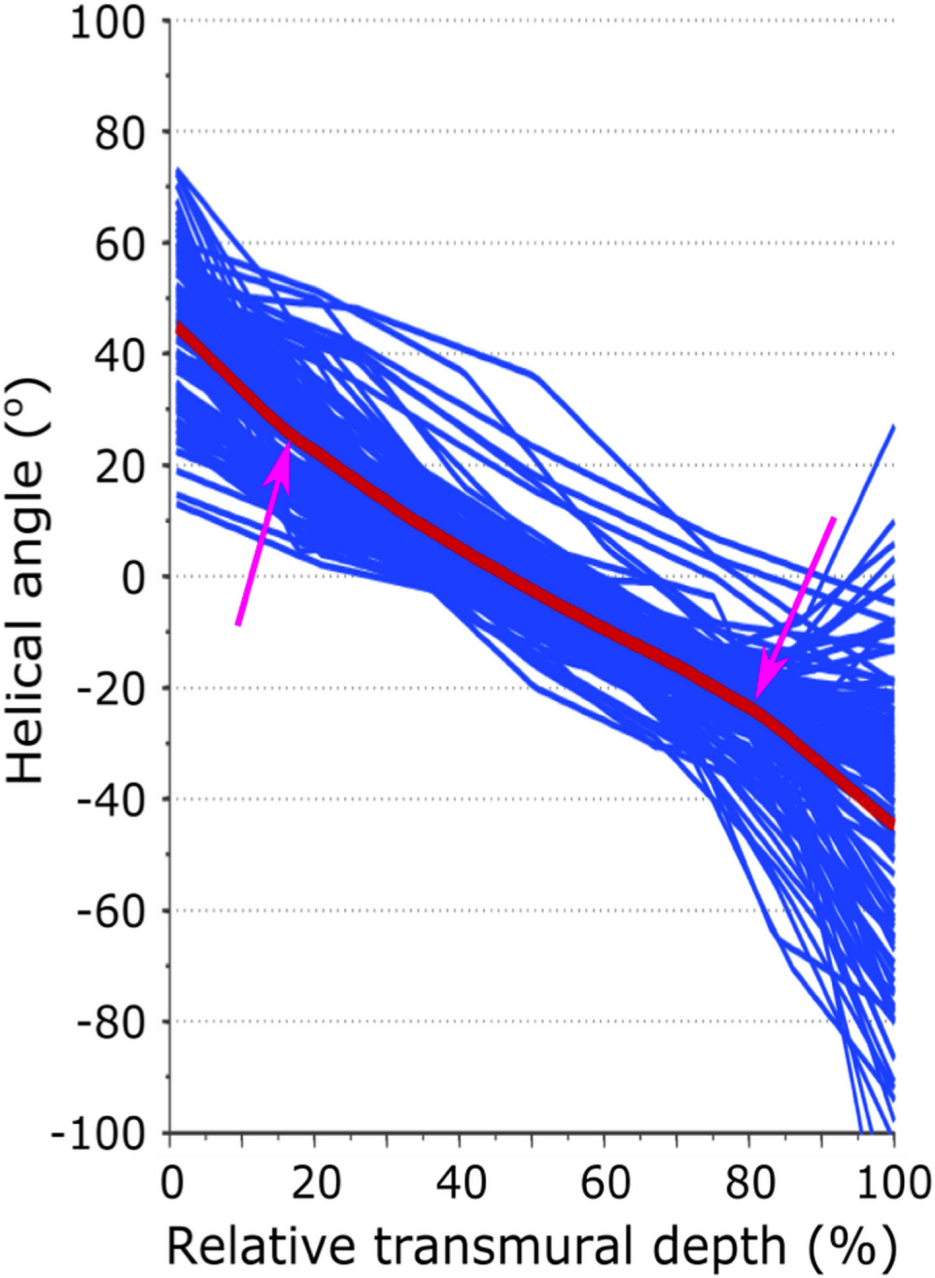
Example of regional HA line profiles. The x-axis shows the percentage distance from the endocardial surface. A relative reduction in the transmural helical angle gradient can be appreciated in the mesocardium.

An example HA map is shown in Fig 1. A colour coded Bullseye plot of transmural and regional helical values is shown in Fig 2. All subjects showed the expected transmural progression of average myocyte orientation from a left-handed helix in the epicardium, to circumferential in the mesocardium, to a right-handed helix in the endocardium. The average myocardial HA gradient was 9.1 ±1.1°/mm. Fig 3 shows the average regional HA line profiles for an example subject. Within the region of the mesocardium there is a subtle reduction in the rate of change of HA compared to the adjacent transmural zones.

### Fractional anisotropy

**Fig 4:**
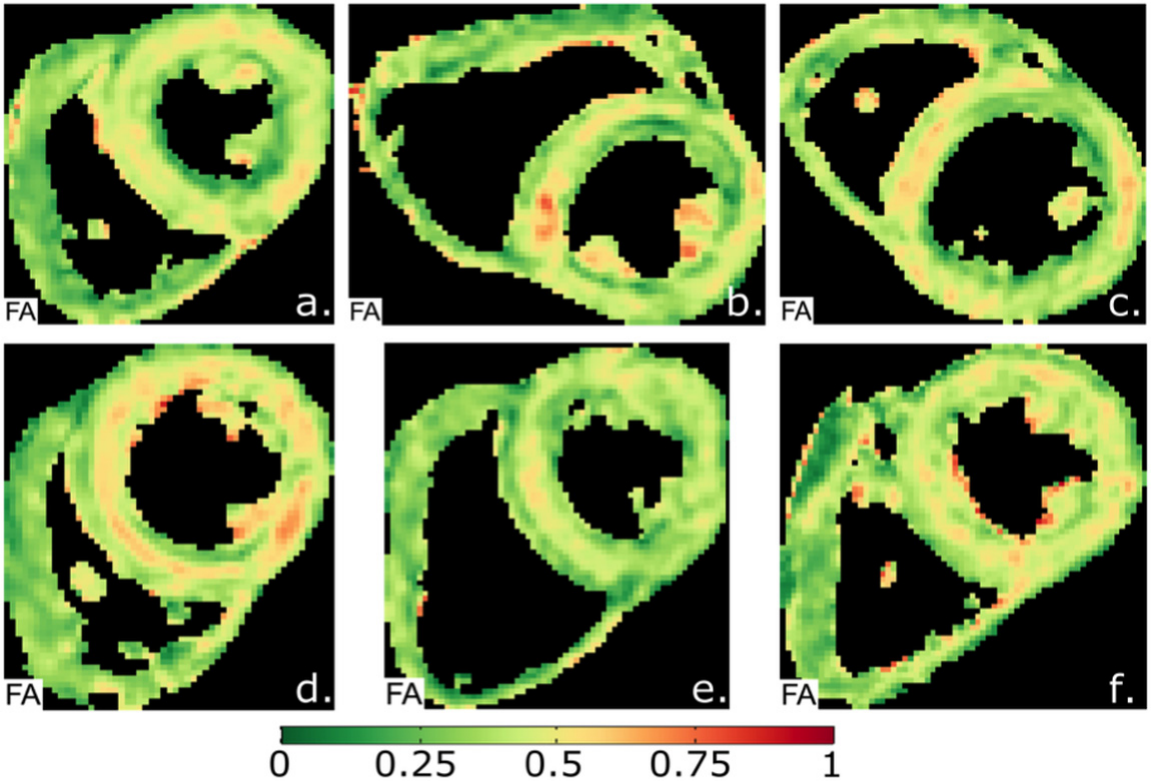
Typical examples of fractional anisotropy (FA) maps. The maps show a circumferential increase in FA (red) in the mesocardium, indicating a more anisotropic composition of myocytes compared to the endo- and epicardium.

**Fig 5:**
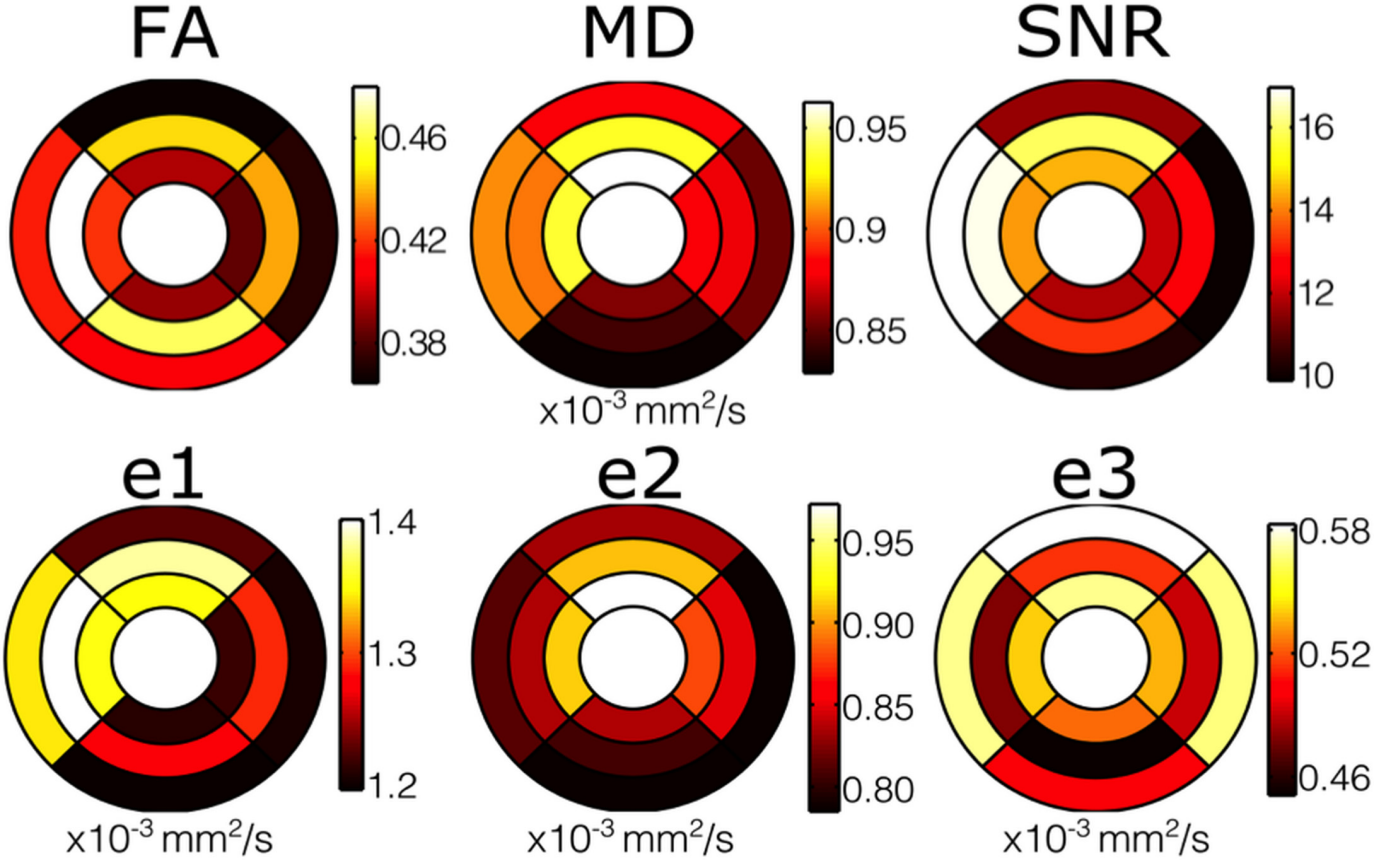
Colour Bullseye maps showing the significant heterogeneity in the distribution of FA, MD, SNR and e1-e3. The outer ring shows results from the epicardium, the middle ring shows the mesocardium, and the inner ring shows the endocardium of the single left ventricular slice. The four walls are also shown in their usual positions: Upper- anterior wall; left- septum; lower- inferior wall; right- lateral wall.

The average global FA per subject was 0.42 ±0.03. Example quantitative FA maps are shown in Fig 4, which in 19 of 20 subjects showed circumferentially increased FA (yellow/orange) but most marked in the septal mesocardium. On transmural analysis, the FA in the mesocardium (0.46 ±0.04) was greater (more anisotropic) than the endocardium (0.40 ±0.04, p≤0.001) and the epicardium (0.39 ±0.04, p≤0.001) Fig 5. There was no difference between endocardial and epicardial FA (p = 1.0). Results for analysis of FA by LV wall are shown in Table 1. With the lateral wall as the reference (0.40 ±0.05), there was no significant difference in FA between the anterior or inferior walls. In contrast, the FA in the septum was significantly greater (0.44 ±0.03, p = 0.04).

**Table 1:** Fractional Anisotropy Regional Analysis. Lateral wall used as the reference for statistical comparisons.

FA	N	Mean	SD	Difference	95% Confidence Interval*	p value*
LV wall						
Lateral	20	0.40	0.05	Reference		
Anterior	20	0.40	0.04	-0.004	-0.030, 0.022	1.0
Inferior	20	0.42	0.05	-0.025	-0.054, 0.003	0.11
Septal	20	0.44	0.03	-0.042	-0.072, -0.012	0.04

*Bonferroni corrected

### Mean Diffusivity

**Fig 6:**
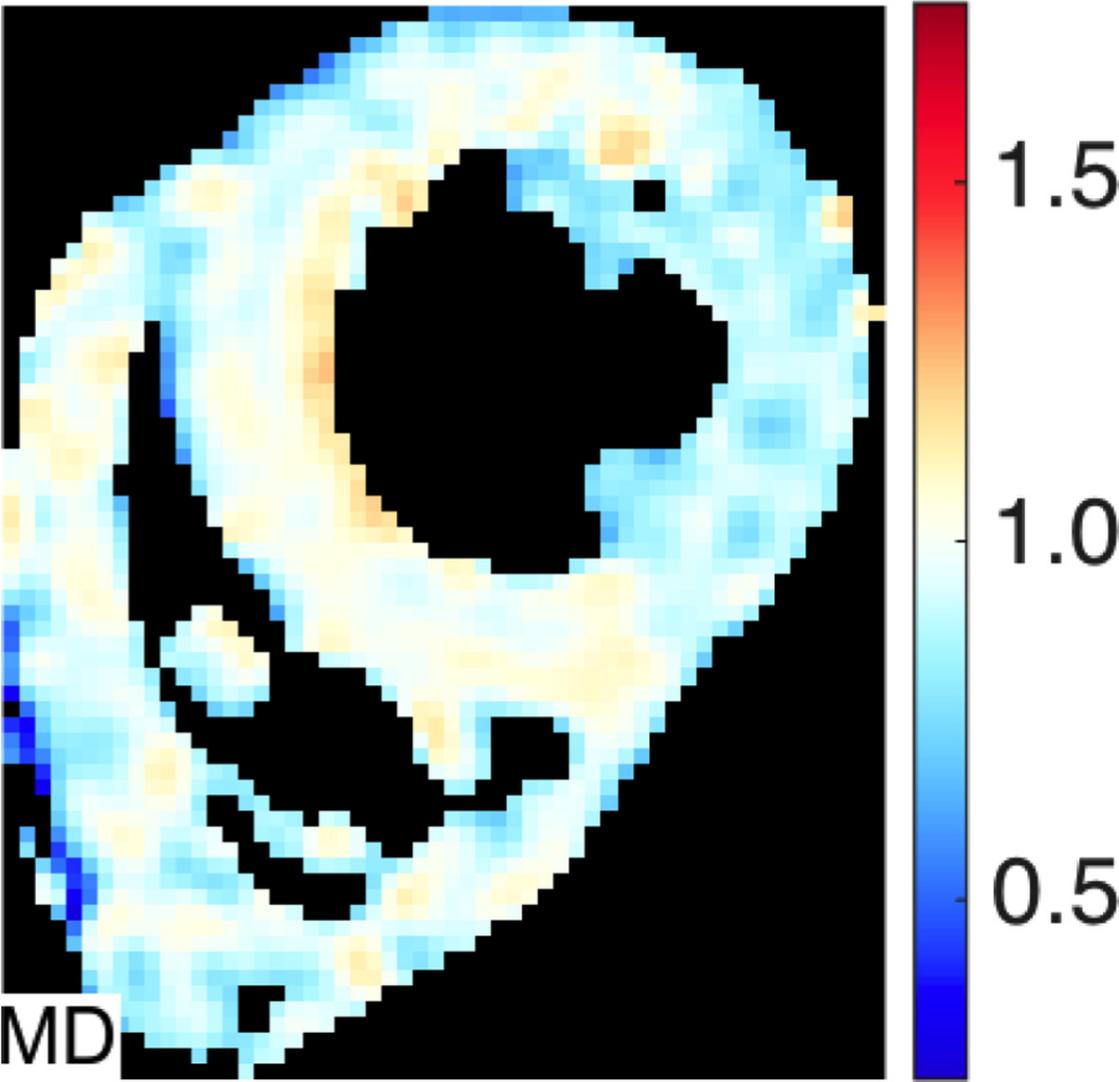
Typical Mean Diffusivity (MD) map. There is a subtle increased in the septal MD compared to the other regions.

The global myocardial MD (all values ×10^-3^mm^2^/s) per subject was 0.89 ±0.06. An example quantitative MD map is shown in Fig 6. Quantitative analysis showed an increase in MD transmurally (epicardium 0.87 ±0.07; mesocardium 0.89 ±0.07; endocardium 0.91 ±0.08) with significantly greater MD in the endocardium compared to the epicardium (p = 0.04, Fig 5). Results for regional analysis of MD are included in table 2. With the lateral wall as the reference (0.87 ±0.08), MD was greater in the anterior wall (0.92 ± 0.07, p = 0.016) and septum (0.92 ±0.07, p = 0.028).

**Table 2:** Mean Diffusivity Regional Analysis (×10^-3^mm^2^s^-1^) Lateral wall used as the reference for statistical comparisons.

MD	N	Mean	SD	Difference	95% Confidence Interval*	p value*
LV wall						
Lateral	20	0.87	0.08	Reference		
Anterior	20	0.92	0.08	-0.057	-0.105, -0.008	0.016
Inferior	20	0.84	0.08	0.026	-0.024, 0.076	0.89
Septal	20	0.92	0.07	-0.047	-0.091, -0.004	0.028

*Bonferroni corrected

### Eigenvalues

The global myocardial eigenvalues (all values in x10^-3^ mm^2^/s) were: e1 = 1.3 ±0.07, e2 = 0.85 ±0.08 and e3 = 0.53 ±0.05. The e1 in the mesocardium (1.33 ±0.08) was greater than in the endocardium (1.28 ±0.09, p = 0.001) and epicardium (1.24 ±0.07, p<0.001), and the e1 in the endocardium was greater than in the epicardium (p = 0.026, Fig 5). There was a transmural gradient in e2 from epicardium (0.80 ±0.07) to mesocardium (0.85 ±0.10; p = 0.001) and endocardium (0.90 ±0.08 p = <0.001). e3 was smaller in the mesocardium (0.48 ±0.06) compared to the endocardium (0.54 ±0.07, p<0.001) and the epicardium (0.55 ±0.07, p<0.001), with no difference between endocardium and epicardium (p = 1.0). Results for regional analysis of the eigenvalues e1, e2 and e3 are included in tables 3, 4 and 5 respectively. With the lateral wall (1.24 ±0.09) as the reference, e1 was greater in the anterior wall (1.32 ±0.10, p = 0.02) and septum (1.37 ±0.08, p<0.001). Taking the lateral wall (0.83 ±0.09) as the reference, e2 was greater in the anterior wall (0.90 ±0.09, p = 0.005). There were no other statistically significant regional differences in measurements of eigenvalues were detected.

**Table 3:** E1 Regional Analysis (×10^-3^mm^2^s^-1^). Lateral wall used as the reference for statistical comparisons.

E1	N	Mean	SD	Difference	95% Confidence Interval*	p value*
LV wall						
Lateral	20	1.24	0.09	Reference		
Anterior	20	1.32	0.10	-0.080	-0.151, -0.010	0.019
Inferior	20	1.23	0.10	0.016	-0.054, 0.086	1.0
Septal	20	1.37	0.08	-0.13	-0.084, 0.002	≤0.001

*Bonferroni corrected

**Table 4:** E2 Regional Analysis (×10^-3^mm^2^s^-1^). Lateral wall used as the reference for statistical comparisons.

E2	N	Mean	SD	Difference	95% Confidence Interval*	p value*
LV wall						
Lateral	20	0.83	0.09	Reference		
Anterior	20	0.90	0.09	-0.067	-0.070, 0.014	0.005
Inferior	20	0.81	0.10	0.027	-0.026, 0.079	0.91
Septal	20	0.85	0.09	-0.016	-0.071, 0.039	1.0

*Bonferroni corrected

**Table 5:** E3 Regional Analysis (×10^-3^mm^2^s^-1^). Lateral wall used as the reference for statistical comparisons.

E3	N	Mean	SD	Difference	95% Confidence Interval*	p value*
LV wall						
Lateral	20	0.53	0.08	Reference		
Anterior	20	0.55	0.08	-0.023	-0.075, 0.029	1.0
Inferior	20	0.49	0.08	0.041	-0.014, 0.095	0.24
Septal	20	0.53	0.05	0.002	-0.042, 0.046	1.0

*Bonferroni corrected

### SNR

The average global myocardial SNR measured in the reference images was 13.2 ±2.2. The SNR was greater in the mesocardium (14.5 ±2.5) than the endocardium (13.0 ±2.2, p<0.001), and epicardium (12.0 ±2.4, p<0.001, Fig 5). The results for regional wall analysis are included in table 6. With the lateral wall (11.5 ± 1.5) as the reference, the SNR was greater in the septum (16.1 ± 3.4, p<0.001) and anterior wall (14.0 ± 3.1, p<0.001).

**Table 6:** SNR Regional Analysis. Lateral wall used as the reference for statistical comparisons.

SNR	N	Mean	SD	Difference	95% Confidence Interval*	p value*
LV wall						
Lateral	20	11.5	1.5	Reference		
Anterior	20	14.0	3.1	-2.58	-4.10, -1.10	≤0.001
Inferior	20	11.7	2.3	-0.284	-1.33, 0.76	1.0
Septal	20	16.1	3.4	-4.64	-6.34, -2.94	≤0.001

*Bonferroni corrected

### Higher Resolution Imaging

**Fig 7:**
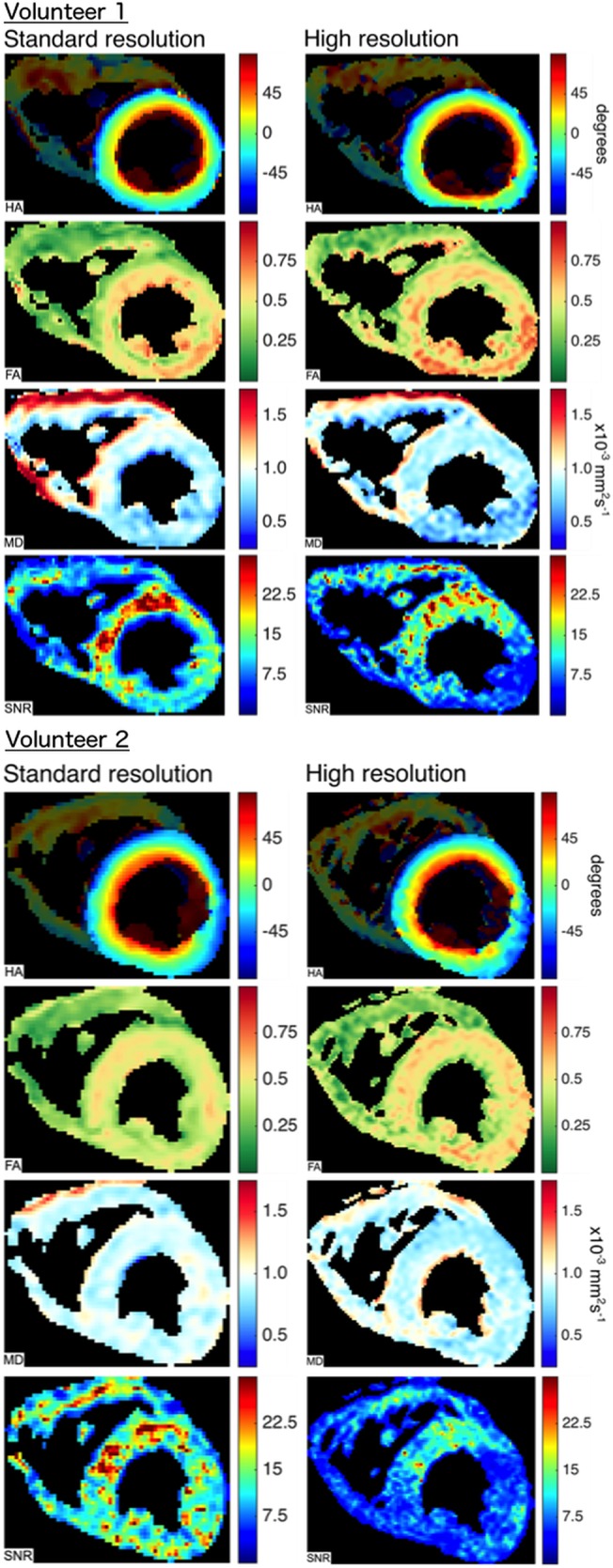
HA, FA, MD and SNR maps for 2 subjects at standard resolution (2.8x2.8x8mm^2^) and higher resolution (1.9x1.9x6mm^2^). In both cases the SNR is reduced in the higher resolution scan, especially in the lateral and inferior walls. There is no difference in the transmural helical angle patterns. In regions of poor SNR in the higher resolution scans, the FA is increased and the MD is decreased. Transmurally there is little change in the FA heterogeneity between standard and higher resolution imaging.

Comparative HA, MD, FA & SNR maps for both standard resolution and high resolution scans for 2 volunteers are shown in Fig 7. In both cases, the SNR maps show lower transmural SNR in the higher resolution images (global SNR 11.5 vs 15.4 in subject 1 and 8.5 vs 14.8 in subject 2), especially in the inferior and lateral walls, with worsening of the inferior wall susceptibility artefact. There was also a relative decrease in myocardial MD with the higher resolution sequence (0.82 vs 0.88 and 0.88 vs 0.93 in the two subjects) and an increase in FA (0.55 vs 0.48 and 0.51 vs 0.46). In both cases the relative increase in mesocardial FA is present at both resolutions. In regions of poor SNR, the FA is elevated.

## Discussion

### Overall findings

This cDTI study demonstrates significant transmural heterogeneity of in-vivo values of fractional anisotropy (FA) and mean diffusivity (MD) in normal human hearts. In keeping with our previous work [7], regional variations in MD were also observed, however the additional regional variation in FA is a new observation. The findings indicate that quantification of FA for clinical purposes, such as the potential use of FA to assess the myocardium for microstructural abnormalities (such as disarray) in hypertrophic cardiomyopathy, must take into account the location and transmural extent of the region of interest from which the measurement is made. The ability to resolve the transmural variation in FA has resulted from the optimisation of cDTI which has improved the sensitivity to diffusion [26].

While the progression in helical angle (HA) is well known from both ex-vivo DTI [4,35,37], and in-vivo cDTI studies [2,4,6–8], and the FA values obtained in this work are consistent with the global values obtained in our previous work [8,26,38], the MD values are lower than those obtained in published data from Nielles-Vallespin et al. [6], (1.14 ±0.15 x10^-3^ mm^2^/s) and Tunnicliffe et al. [8], (1.10 ±0.06 x10^-3^ mm^2^/s). This can be attributed to the higher b_main_ value of 750 s/mm^2^ in this study, compared to b_main_ 350 x 10^−3^ mm^2^/s in prior work [6,8], combined with a b_ref_ value of 150 s/mm^2^. While this protocol was found to be optimal for limiting the contribution from microvascular perfusion, one consequence of these changes is a difference in the measured cDTI parameters, including a reduction in the measured MD [26].

### Influence of microstructure on FA and MD measurements

As measured in vivo by cDTI, multiple factors are likely to contribute to FA values, including microstructural anisotropies, microstructural dynamics and possible artefacts associated with acquisition in the beating heart. Of the e1, e2 and e3 components of diffusion, the combination of high mesocardial e1 and low e3, would elevate FA in this layer. At the same time, there was an increase of e2 from epicardium to endocardium, at least in the late systolic phase investigated. One potential microstructural contributor to the FA changes could be a transmural variation in HA gradient. While the transmural variation in HA makes a layer-wise assessment of HA difficult, we did observe a small reduction in mesocardial HA gradient, which accords with the findings of ex-vivo DTI studies in canine [2] and human hearts [29].^,^ Mesocardial e1 may therefore be elevated through a relative increase in intravoxel e1 alignment. This hypothesis is supported by histological studies which have documented a plateau in myocyte angulation within circumferentially orientated mesocardial myocytes [39,40]. Jiang et al. previously reported transmural heterogeneity of FA within sheep myocardium [28]. In contrast to the marked helical arrangement of human sub-epicardial myocytes, sub-epicardial myocytes in sheep are relatively more circumferentially orientated [41]. In line with our work, they similarly detected a relative increase in anisotropy within these circumferential myocytes. This was attributed to the decrease in e2 and e3 in the region, although e1 also increased but did not reach significance. One possible reason for the larger changes we observed in e1 is the longer mixing time of the DW-STEAM sequence used in our study when compared to the spin-echo sequence used by Jiang et al [28].

In addition to the well-known arrangement of myocytes from a left to a right-handed helical angle, the myocytes are also arranged in small functional units or sheetlets, separated by shear layers [42]. Sheetlets are thought to cyclically re-orientate throughout the cardiac cycle, with the shearing of the thin extracellular layers between adjacent sheetlets. This is understood to contribute to the considerable radial myocardial thickening observed in vivo [43]. LeGrice et al. measured a transmural increase in extracellular relative to intracellular volume towards the endocardium in carefully prepared canine myocardial specimens [44], and histological studies have shown the relative absence of shear layers epicardially [42,45,46].The effects of these laminar structures on diffusion parameters have yet to be elucidated in humans, but if, as might be expected, diffusion along shear layers contributes to e2 and possibly e3, then any increase in their abundance might explain the increase in e2 from epi- to endocardium, also described by Jiang et al. [28], and also the MD gradient from epi- (lowest) to endocardium. However, the reduced e3 in the mesocardium is more difficult to explain on this basis, although it could be a reflection of a more coherent laminar structure in this layer, for instance if there was greater dominance of one, or a smaller angle between two laminar populations, as predicted in some simulations [46]. Studies documenting the electrophysiological properties of the ventricular myocardium add further support for the heterogeneity of the transmural myocardium [47]. The significant electrical differences between transmural and longitudinal cell layers may result from propagation boundaries imposed by the varying complex cellular architecture.

### Influence of strain on FA and MD measurements

Quantitative cDTI parameters acquired with monopolar sequences must be interpreted in the context of myocardial strain, which has been reported to interact with diffusion encodings [20–22]. Myocardial strain, as measured macroscopically, is maximal in the radial, cross myocyte direction and increases transmurally from the epicardium to endocardium [48]. As measurements of cDTI in this study were performed in systole, this could contribute to the gradient observed in e2, which reflects cross myocyte diffusions, however this would not appear to account for the mesocardial depression of e3 and elevation of e1 and FA. Strain could also modify the MD measurement, and there is evidence for higher measured MD in systole compared with diastole [8,49]. The transmural heterogeneity of strain may also have contributed to the observed increase in endocardial MD [48].

Cardiac DTI can be acquired without strain effects. One approach is the bipolar spin echo sequence [5], however the short mixing time between winding and unwinding gradients, allows limited time for diffusion through the complex myocardial microstructure; mitigating the effects of motion during the long diffusion gradients is also challenging. An alternative strategy is to acquire cDTI data within the strain ‘sweet spot’ [21,22], but in practice thi is difficult to define; image quality is degraded by myocardial motion; and the myocardium is comparatively thinner than systole, therefore reducing the number of pixels across the LV wall. A further option would be to undertake strain correction of the diffusion tensor; however current techniques are limited, as they make the assumption that the myocardium is an isotropic jelly like material [21,22, 50–53]. This point was illustrated in recent work by Stoeck et al. [53], which presented strain corrected cDTI data in systole and diastole acquired with the DW-STEAM EPI sequence. Post strain correction, the difference between systolic and diastolic MD appeared to increase, however as this parameter reflects the average diffusivity throughout the cardiac cycle, one would have expected the correction to eliminate the difference. [53].

### Influence of SNR on FA and MD measurements

We observed that increasing values of SNR were associated with higher values of FA both transmurally and regionally. The influence of SNR on FA and MD is difficult to predict; at low b-values, noise results in an overestimation of FA [54,55] whereas at high b-values, noise results in an underestimation of both FA and MD [54]. The transition from the low to high b-value regime depends on a number of factors including both the FA and MD of the tissue and the SNR of the acquisition. While previous work has studied these effects in the brain, and for diffusion weighted imaging generally [56], a comprehensive set of simulations with myocardium specific parameters is required to fully characterise this transition in the heart. However, in the high b-value regime, we would expect to observe that higher measurement noise levels, with lower SNR, would favour lower anisotropy. Our data showed lower FA and SNR in the epicardium & endocardium, thus we might conclude that our sequence is a high b-value regime for FA, and that SNR contributes to transmural FA heterogeneity. However, in contradiction to this possible conclusion, is that the FA maps at higher resolution (Fig 6) appear to demonstrate an increase in FA in areas of low SNR, in keeping with a low b-value regime. The observed relationship of decreasing MD regionally with low SNR is also in keeping with a high b value regime for MD. The MD pattern does not track SNR transmurally however, and other factors appear to influence these MD measurements.

### Influence of resolution and partial volume effects on FA and MD measurements

A further potential contribution to heterogeneity of FA and MD measurements is from partial volume effects, which would be most marked in the endocardium and epicardium. The potential influence could be through direct measurement aberration but also through reduced SNR. The relatively low resolution of our sequence (six pixels acquired resolution on average across the septum) may thus have contributed to the observed heterogeneity. In an attempt investigate this further we performed higher resolution scans in 2 subjects who were able to comply with the very long breath holds required (average 40s). The FA maps showed little difference in the pattern of heterogeneity between standard and high resolution scanning, arguing against partial voluming as a significant contributor however more subject data is required to confirm this quantitatively.

Our data demonstrates that the trade off with higher resolution is reduced SNR and increased susceptibility to artefact; this, combined with need for either prohibitively long or additional breath holds, limits the clinical application of higher resolution STEAM cDTI presently.

## Conclusions

The above consideration of factors that might influence FA and MD allows their relative non-specificity as parameters for the characterisation of myocardial structure to be appreciated. More differentiated analysis of cDTI, for example by distinguishing and appropriately analysing the values of each of the three eigenvectors of diffusion, may therefore be advantageous, although interpretation is likely to remain challenging. Using an optimised in-vivo cDTI sequence in normal subjects, we have shown significant transmural and regional variation in MD and particularly FA, which indicates that quantification of this measurement requires careful selection of the region of interest. Standardisation of the measurement method of FA and MD would be required to interpret values between studies and centres, and the possible interpretation of the presence of abnormal myocardium on a microstructural level, such as disarray, would require demonstration that the magnitude of the quantitative differences versus normal significantly exceeds these normal variations.

## Supporting Information

S1 File Workbook of analysed cDTI data: Excel workbook of analysed regional and transmural FA and MD data. (XLSX)
